# Datopotamab deruxtecan induces hallmarks of immunogenic cell death

**DOI:** 10.15698/cst2025.08.311

**Published:** 2025-08-11

**Authors:** Sabrina Forveille, Marion Leduc, Allan Sauvat, Guido Kroemer, Oliver Kepp

**Affiliations:** 1 Metabolomics and Cell Biology Platforms, Gustave Roussy Cancer Center, Université Paris Saclay, Villejuif, France.; 2 Centre de Recherche des Cordeliers, Equipe labellisée par la Ligue contre le cancer, Université Paris Cité, Sorbonne Université, Inserm U1138, Institut Universitaire de France, Paris, France.; 3 Institut du Cancer Paris CARPEM, Department of Biology, APHP, Hôpital Européen Georges Pompidou, Paris, France.

**Keywords:** anticancer immunotherapy, antibody drug conjugate, bystander effect

## Abstract

Antibody-drug conjugates (ADCs) offer a strategy for targeted delivery of cytotoxic agents to cancer cells. In this study, we investigated the mechanism of action of datopotamab deruxtecan, an ADC composed of a monoclonal antibody targeting tumor-associated calcium signal transducer 2 (TACSTD2, also known as trophoblast cell-surface antigen-2 (TROP2)) conjugated to the topoisomerase I inhibitor DXd. Datopotamab deruxtecan reduced the viability of human osteosarcoma U2OS cells engineered to express TROP2, but had no effect on their parental counterparts, which only expressed the CALR-GFP biosensor. In TROP2-expressing cells, it triggered the translocation of CALR-GFP from the ER to the cell periphery. Both datopotamab deruxtecan and its DXd payload elicited several features characteristic of immunogenic cell death (ICD), including detectable calreticulin exposure on the cell surface, release of high-mobility group box 1 (HMGB1), and ATP secretion into the culture medium. Importantly, the TROP2-targeted ADC also exerted a bystander antitumor effect on parental U2OS cells (lacking TROP2 expression) co-cultured with TROP2-expressing U2OS cells. These findings demonstrate that datopotamab deruxtecan delivers a cytotoxic payload capable of inducing hallmark features of ICD *in vitro*.

## Abbreviations

ADCs - antibody-drug conjugates,

DAMPs - danger-associated molecular patterns,

DCs - dendritic cells,

HR - hormone receptor,

ICD - immunogenic cell death,

ICIs - immune checkpoint inhibitors,

ISO - isotype control,

NSCLC - non-small cell lung cancer.

## INTRODUCTION

Anticancer therapies, including chemotherapies, targeted agents, and antibody-drug conjugates (ADCs), achieve durable efficacy *in vivo* only when they combine direct tumor cell killing with stimulation of the immune system 1,2. This immune-mediated component often involves the induction of immunogenic cell death (ICD), a regulated form of cell death that promotes T cell-mediated responses against residual cancer cells 3. ICD is defined by a coordinated series of stress responses that lead to the emission of danger-associated molecular patterns (DAMPs), which serve as adjuvants to activate dendritic cells (DCs) and support antigen presentation 4,5,6. These DAMPs include autophagy-dependent ATP secretion, ER stress-induced calreticulin (CALR) exposure, and HMGB1 release. ATP acts as a chemoattractant for cDC1s via P2RY2, CALR engages LRP1 to facilitate phagocytic uptake, and HMGB1 promotes DC maturation through TLR4 7,8. Moreover, ICD inducers often sensitize tumors to sequential immune checkpoint blockade 2,9,10.

ADCs provide a means to selectively deliver cytotoxic payloads to tumor cells while sparing healthy tissues 11. Notably, a number of ADC payloads, including DXd, maytansinoids, auristatins, and pyrrolobenzodiazepines, have been shown to induce ICD 12,13,14,15,16,17,18,19. Non-small cell lung cancer (NSCLC) accounts for the majority of lung cancers, with EGFR mutations present in up to 38% of patients 20. Many patients present with advanced disease, and although EGFR TKIs and platinum-based chemotherapy offer initial benefits, resistance frequently emerges 21. Here, we evaluated the immunogenic potential of datopotamab deruxtecan, an ADC composed of a human anti-TROP2 IgG1 monoclonal antibody conjugated to multiple molecules of the topoisomerase I inhibitor DXd via a cleavable tetrapeptide linker. In clinical trials, datopotamab deruxtecan has shown durable responses in patients with advanced NSCLC who progressed on EGFR TKIs and chemotherapy, leading to its recent accelerated FDA approval 22. Moreover, datopotamab deruxtecan also received FDA approval for the treatment of unresectable or metastatic, hormone receptor (HR)-positive, HER2-negative breast cancer 23. In this study, we demonstrate that datopotamab deruxtecan induces hallmark features of ICD *in vitro*, supporting its dual role as a direct cytotoxic agent and potential stimulator of anticancer immunity.

## RESULTS

### Datopotamab deruxtecan reduces viability and induces ICD traits in TROP2^+^ cells

**Figure 1:**
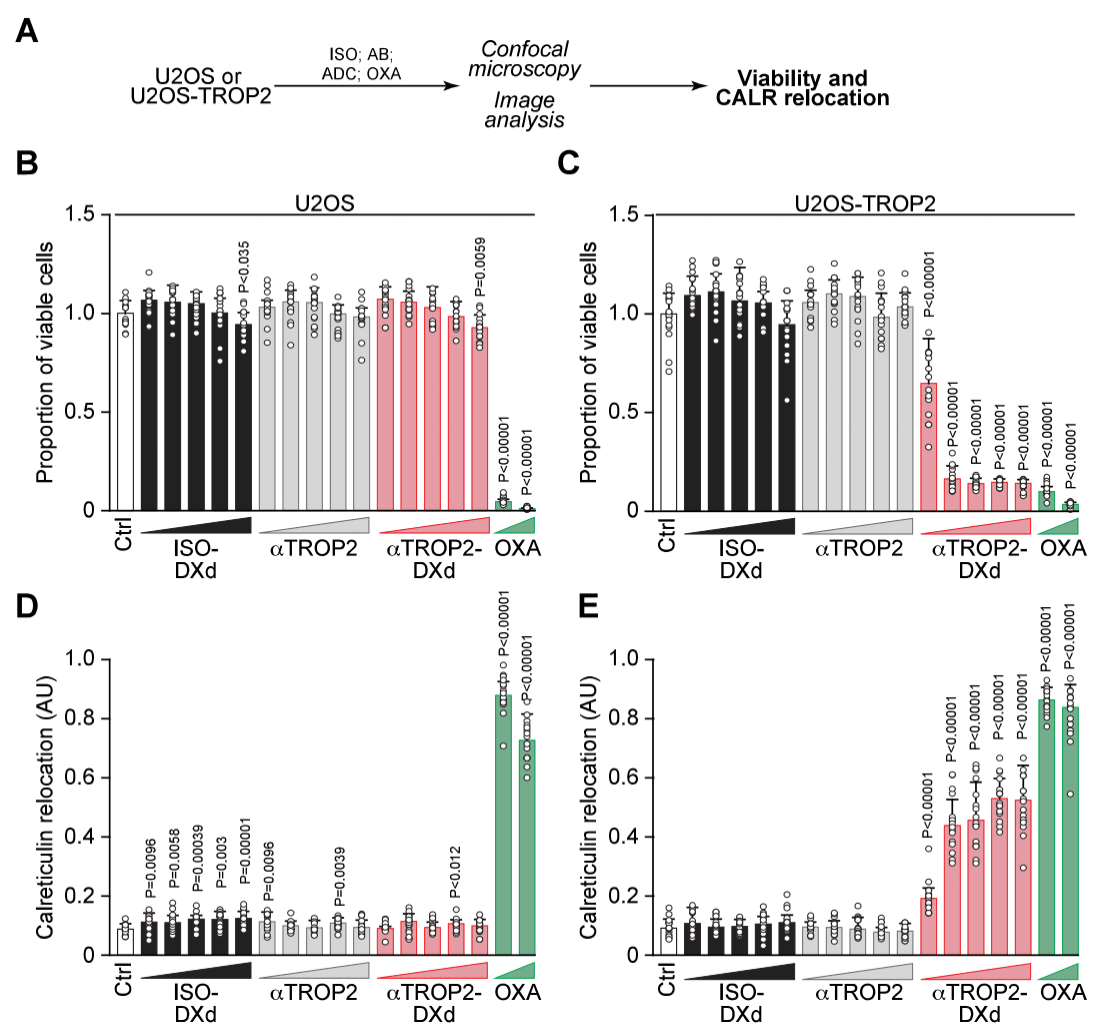
FIGURE 1: Datopotamab deruxtecan reduces viability and promotes CALR relocalization in TROP2-expressing cells. **(A)** Schematic representation of the treatment and analysis workflow. **(B-D)** U2OS osteosarcoma cells stably expressing CALR-GFP and their TROP2-overexpressing counterparts were treated for 5 days with increasing concentrations of a non-targeting IgG-DXd control ADC (ISO), anti-TROP2 monoclonal antibody (datopotamab), or the TROP2-targeted ADC datopotamab deruxtecan (ADC) across a dose range of 0.04-10 µg/mL. Oxaliplatin (OXA; 50 µM) served as a positive control for ICD induction. **(B, C)** Cell viability was determined by fluorescence microscopy, expressed relative to untreated controls. **(D, E)** Quantification of GFP signal heterogeneity was used to assess CALR redistribution. Data are shown as mean ± SD of quadruplicates, normalized to untreated controls. Individual data points represent single imaging fields. Statistical significance was calculated using Welch’s t-test.

Datopotamab deruxtecan, a TROP2-targeting ADC incorporating the exatecan-derived pyranoindolizinoquinoline DXd, was evaluated for its cytotoxic and immunogenic effects. Prior studies have shown that compounds in this family can trigger ICD and promote anticancer immunity *in vivo* 24,25,26. To assess its mode of action, we used U2OS osteosarcoma cells, a validated *in vitro* model for ICD characterization 12. These cells were engineered to express TROP2 via lentiviral transduction. Viability was determined by epifluorescence microscopy, excluding pyknotic nuclei identified by Hoechst 33342. Datopotamab deruxtecan reduced viability in a TROP2-dependent, dose-responsive manner, while parental U2OS cells remained unaffected at equivalent concentrations. Neither the unconjugated anti-TROP2 antibody (datopotamab) nor a non-targeting IgG-DXd isotype control (ISO) induced cytotoxicity in either cell type, underscoring the importance of the DXd payload (**Fig. 1A-C**). At 5 days post-treatment, viability loss was pronounced in TROP2^+^ CALR-GFP biosensor cells treated with datopotamab deruxtecan but not in TROP2^-^ controls. By contrast, oxaliplatin decreased viability in both cell populations (**Fig. 1A-C**). Using CALR-GFP-expressing cells, we also evaluated the subcellular localization of CALR. Datopotamab deruxtecan caused a peripheral redistribution of CALR-GFP in TROP2^+^ cells, resembling the phenotype induced by oxaliplatin, while no such effect was observed in TROP2^-^ cells (**Fig. 1D-E**), suggesting that the ADC induces immunogenic CALR exposure.

### Datopotamab deruxtecan triggers hallmarks of ICD

**Figure 2:**
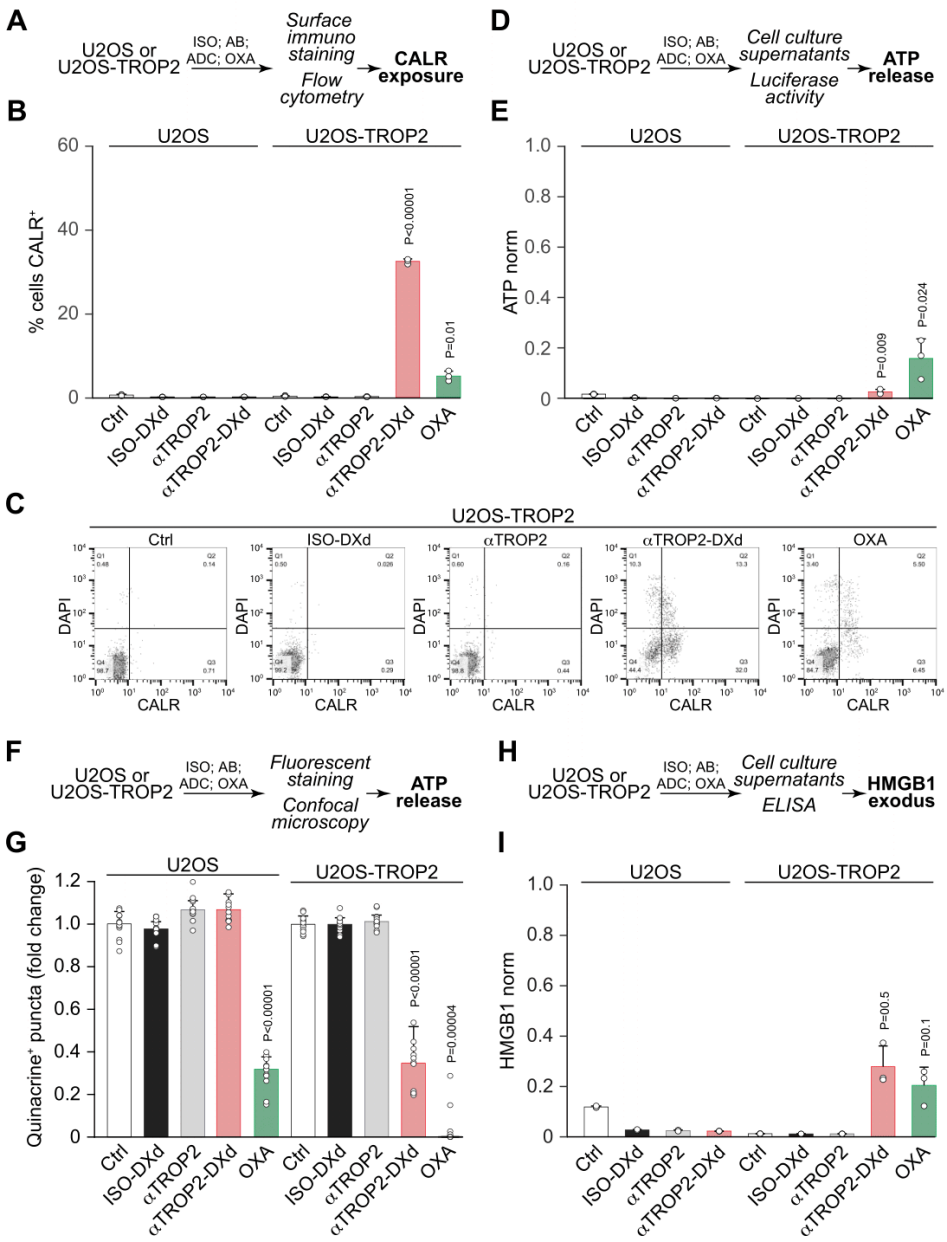
FIGURE 2: Datopotamab deruxtecan induces ICD hallmarks in TROP2^+^ but not parental U2OS cells. TROP2^+^ and parental U2OS cells were treated for 5 days with 0.16 µg/mL of ISO, datopotamab, or datopotamab deruxtecan (ADC). OXA (5 µM) and mitoxantrone (MTX, 10 nM) served as positive ICD controls. Parental cells were also treated with 0.16 µg/mL of free DXd. **(A-C)** Surface CALR exposure was assessed via immunostaining and flow cytometry; data show the percentage of DAPI^-^/CALR^+^ viable cells. **(D, E)** Extracellular ATP levels were quantified from supernatants using a luciferase-based bioluminescence assay. **(F, G)** Intracellular ATP-containing vesicles were assessed by quinacrine staining. **(H, I)** HMGB1 release into culture supernatants was measured by ELISA. Data represent mean ± SD of triplicate samples, normalized to cell number per microscopy. Statistical significance determined by Welch’s t-test.

We next assessed additional ICD markers in TROP2^+^ and parental U2OS cells treated with the ADC, datopotamab alone, or ISO. Flow cytometric analysis of viable (DAPI^-^) cells revealed that datopotamab deruxtecan significantly increased surface CALR exposure exclusively in TROP2^+^ cells (**Fig. 2A-C**). It also induced ATP release, as indicated by luciferase-based detection in supernatants (**Fig. 2D, E**) and confirmed by the loss of quinacrine-positive intracellular vesicles (**Fig. 2F, G**). Moreover, datopotamab deruxtecan stimulated the release of HMGB1 from TROP2^+^ cells, as detected by ELISA (**Fig. 2G, H**).

### Bystander killing in mixed cultures

**Figure 3:**
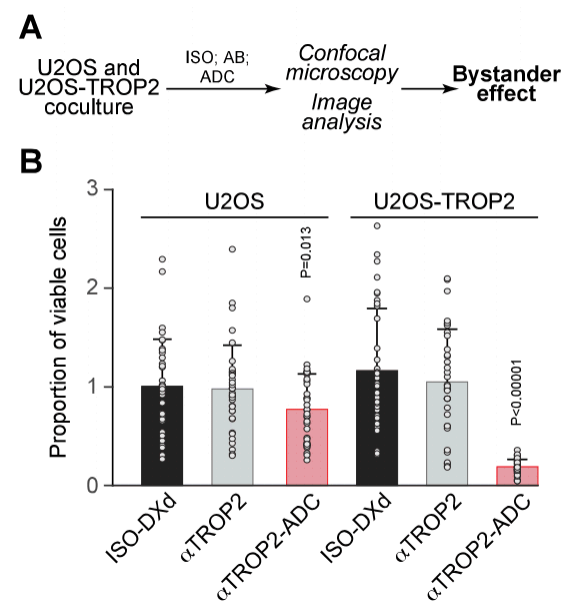
FIGURE 3: Datopotamab deruxtecan exerts bystander cytotoxic effects in cocultures of TROP2^+^ and parental U2OS cells. (**A**) Schematic overview of the coculture assay. (**B**) Parental and TROP2^+^ U2OS cells labeled with distinct fluorescent markers were mixed 1:1 and treated for 5 days with 0.16 µg/mL of ISO, datopotamab or datopotamab deruxtecan (ADC). Viability was assessed separately for each population using fluorescence microscopy and expressed relative to untreated controls. Results are shown as mean ± SD of quadruplicate samples. Data points represent individual view fields. Significance was assessed using Welch’s t-test.

Finally, we explored potential bystander effects by coculturing TROP2^+^ and TROP2^-^ U2OS cells at a 1:1 ratio. While TROP2^-^ cells were unaffected in monocultures (**Fig. 1A-C**), their viability was significantly reduced when cocultured with TROP2^+^ cells treated with datopotamab deruxtecan (**Fig. 3**). These results indicate that the ADC can mediate cytotoxic effects on neighboring TROP2^-^ cells, supporting the existence of a bystander antitumor effect.

## DISCUSSION

This study demonstrates that datopotamab deruxtecan, an ADC targeting TROP2, induces ICD in human cancer cell lines *in vitro*. In parallel, the topoisomerase I inhibitor payload DXd, derived from a pyranoindolizinoquinoline scaffold and structurally related to exatecan, exhibited potent cytotoxicity against murine tumor cells, leading to long-lasting, antigen-specific immune responses. These findings align with previous studies reporting that pyranoindolizinoquinoline-based compounds, administered either systemically or encapsulated in nanocarriers, can promote ICD and enhance responses to immune checkpoint inhibitors (ICIs) in preclinical models 25,27,28.

Datopotamab deruxtecan consists of a fully human IgG1 monoclonal antibody directed against the tumor-associated antigen TROP2 (TACSTD2), conjugated via a tetrapeptide-based cleavable linker to an average of four molecules of DXd. The linker is selectively cleaved by lysosomal proteases after internalization, allowing targeted intracellular delivery of DXd. TROP2 is overexpressed in various epithelial malignancies, including NSCLC, triple-negative breast cancer (TNBC), and urothelial carcinoma, making it an attractive target for ADC therapy 29.

Here, we showed that the unconjugated anti-TROP2 antibody (datopotamab) alone did not reduce the viability of TROP2-overexpressing human U2OS cells, suggesting that these cells are not dependent on TROP2 signaling for survival. Likewise, a non-targeting IgG-DXd isotype control had no cytotoxic effect. In contrast, datopotamab deruxtecan effectively and selectively killed TROP2^+^ cells, while sparing TROP2^-^ cells, indicating a high degree of target specificity and a potentially favorable therapeutic index. In mixed cultures containing both TROP2^- ^and TROP2^+^ cells, datopotamab deruxtecan treatment also reduced the viability of TROP2^-^ cells, an effect consistent with bystander killing. This phenomenon, which is frequently observed with membrane-permeable payloads such as DXd, is mediated by diffusion of the released cytotoxin from targeted cells into the surrounding microenvironment 30,31. Importantly, while Dato-DXd requires TROP2-mediated internalization for activation, the local release of a cytotoxic payload capable of crossing cell membranes allows for broader cytotoxicity within the tumor niche. Such activity can be advantageous in tumors with heterogeneous antigen expression or in cases of antigen loss due to immunoselection. The impact on normal tissues appears limited in clinical settings, suggesting that bystander effects are spatially confined and do not broadly harm non-transformed cells, although further studies are warranted to fully understand tissue-specific responses.

Moreover, ADCs like datopotamab deruxtecan may modulate the tumour immune microenvironment through multiple mechanisms, including the depletion of immunosuppressive cell populations like regulatory T cells (Tregs) and myeloid-derived suppressor cells (MDSCs), which in turn enhances the infiltration, activation, and cytotoxic function of effector CD8^+^ and CD4^+^ T cells; alternatively, the cytotoxic payload, DXd, may directly stimulate DCs, promoting antigen presentation and priming of adaptive immune responses 30,32,33.

Our data show that datopotamab deruxtecan elicits multiple hallmarks of ICD, including surface exposure of CALR, ATP release, and secretion of HMGB1, which together can stimulate dendritic cell activation and T cell priming. This immunostimulatory effect may enhance tumor antigen presentation and support the development of adaptive immunity 34. ICD inducing chemotherapeutics have synergistic potential when combined with ICIs, as indicated in preclinical experiments 6,35,36, as well as in clinical trials 2,37,38,39. Clinical trials are currently evaluating datopotamab deruxtecan in multiple cancer indications, including EGFR-mutant NSCLC (TROPION-Lung01 and TROPION-Lung05), where it has shown promising results even after failure of EGFR TKIs and platinum-based chemotherapy, as well as in breast cancer, where datopotamab deruxtecan shows effect in heavily pretreated advanced HR-positive/HER2-negative and TNBC (TROPION-PanTumor01) 40,41. These observations support further investigation of datopotamab deruxtecan in combination with checkpoint inhibitors (such as ongoing clinical trials targeting NSCL including Phase 1b TROPION-Lung02 and TROPION-Lung04 and Phase 3 TROPION-Lung07, TROPION-Lung08 and AVANZAR; as well as TNBC such as TROPION-Breast03, TROPION-Breast04, TROPION-Breast05) especially in tumors with high TROP2 expression and immunosuppressive microenvironments.

## MATERIALS AND METHODS

### Cell lines and cell culture

Human U2OS osteosarcoma cells were sourced from the American Type Culture Collection (ATCC). Cell culture vessels were obtained from Corning (New York, NY, USA). U2OS cells were maintained in Dulbecco’s Modified Eagle Medium (DMEM; Thermo Fisher Scientific, Waltham, MA, USA) supplemented with 10% fetal bovine serum (FBS; Merck, Darmstadt, Germany), non-essential amino acids (NEAA; Thermo Fisher Scientific), and HEPES buffer (Thermo Fisher Scientific). Cells stably expressing a calreticulin-GFP fusion construct were maintained under selective pressure using 5 µg/mL blasticidin and 100 µg/mL zeocin. To generate co-cultures, TROP2-positive and TROP2-negative U2OS cells were combined at a 1:1 ratio. All cultures were grown at 37°C in a humidified atmosphere containing 5% CO_₂_, and routine testing confirmed cultures were free from mycoplasma contamination.

### Reagents and antibodies

Oxaliplatin was obtained from Accord Healthcare (Ahmedabad, India). Datopotamab deruxtecan (anti-TROP2 ADC), the unconjugated anti-TROP2 monoclonal antibody, and a non-targeting IgG-DXd control ADC were provided by Daiichi Sankyo. The rabbit polyclonal anti-calreticulin antibody (ab2907) was sourced from Abcam (Cambridge, UK), and AlexaFluor^™^ 488-conjugated goat anti-rabbit IgG secondary antibody was purchased from Thermo Fisher Scientific.

### Fluorescence microscopy

U2OS cells, either wild-type or stably expressing CALR-GFP with or without TROP2, were seeded at 500 cells per well in 384-well µclear imaging plates (Greiner Bio-One, Kremsmünster, Austria). After overnight incubation, cells were treated as indicated and maintained for five days. Cells were fixed with 3.7% formaldehyde containing 1 µg/mL Hoechst 33342 for 20 minutes. Images were acquired using an IXMc automated confocal microscope (Molecular Devices, San Jose, CA, USA) equipped with a Lumencor light source, Semrock filters (Rochester, NY, USA), a 16-bit sCMOS camera (Andor, Belfast, UK), and a 20× Plan APO objective (Nikon, Tokyo, Japan). Image analysis was performed using the EBImage package in R.

### Cell viability assay

U2OS cells were seeded at 500 cells per well in 384-well plates and allowed to adhere for 24 h prior to treatment with datopotamab deruxtecan, the unconjugated anti-TROP2 antibody, or the non-targeting IgG-DXd ADC. After 5 days of incubation, cells were fixed with 3.7% formaldehyde (Merck) containing 1 µg/mL Hoechst 33342 (Thermo Fisher) for 20 minutes. Fluorescent images were acquired and analyzed as described above.

### Surface calreticulin staining and flow cytometry

Wild-type U2OS cells were seeded at 2,000 cells per well in 96-well µclear plates (Greiner Bio-One). After treatment and 5 days of incubation, cells were detached, transferred to V-bottom 96-well plates, and pelleted by centrifugation at 200 g for 5 minutes. Cells were resuspended in PBS containing 1% BSA and incubated with anti-calreticulin antibody (1:100) for 30 minutes at 4°C. Following two washes, cells were incubated with AlexaFluor^™^ 488-conjugated secondary antibody (1:1000) for 30 minutes at 4°C, washed again, and stained with 1 µM DAPI (cat. no. 62248, Thermo Fisher Scientific). Flow cytometric analysis was performed using a BD LSR Fortessa (BD Biosciences, Franklin Lakes, NJ, USA). CALR^+^ cells were quantified among DAPI^-^ viable cells.

### Extracellular ATP release

U2OS cells were seeded at 2,000 cells per well in 96-well plates and treated the following day. After 5 days, supernatants were collected, centrifuged (200 g, 5 minutes), and transferred to white-walled 96-well plates. ATP levels were measured using the ENLITEN ATP Bioluminescence Detection Kit (Promega, Madison, WI, USA) according to the manufacturer’s instructions. Luminescence was measured at 560 nm using a SpectraMax i3x plate reader (Molecular Devices) and normalized to cell number.

### Intracellular ATP imaging

Cells were seeded at 2,000 cells per well in 96-well µclear imaging plates, treated the next day, and incubated for 5 days. Cells were then incubated in Krebs-Ringer buffer (5 mM KCl, 125 mM NaCl, 0.7 mM KH_₂_PO_₄_, 1 mM MgSO_₄_, 2 mM CaCl₂, 6 mM glucose, 25 mM HEPES, pH 7.4) containing 5 µM quinacrine and 4 µM Hoechst 33342 for 30 minutes at 37°C 42. After washing, images were captured via automated confocal fluorescence microscopy. Environmental conditions for live imaging were controlled using an Ibidi gas mixer (Gräfelfing, Germany). Image processing was conducted in R using EBImage.

### HMGB1 ELISA

U2OS cells were seeded in 96-well plates (2,000 cells/well), treated the next day, and incubated for 5 days. Cell culture supernatants were collected, centrifuged at 200 g for 5 minutes, and analyzed for HMGB1 content using a commercial ELISA kit (IBL International, Tecan, Männedorf, Switzerland) per the manufacturer’s protocol. Absorbance at 450 nm was recorded on a SpectraMax i3x plate reader, and values were normalized to cell number.

### Statistical analysis

All experiments were performed in triplicate or quadruplicate unless otherwise indicated. Data are presented as mean ± standard deviation (SD). Statistical comparisons were made using Welch’s or Student’s t-test, implemented via the t.test function in R (https://www.r-project.org).

### Data availability

Data are available from the corresponding authors G.K. and O.K. on reasonable request.

## CONFLICT OF INTEREST

O.K. is a scientific co-founder of Samsara Therapeutics. GK has been holding research contracts with Daiichi Sankyo, Eleor, Kalei-do, Lytix Pharma, PharmaMar, Osasuna Therapeutics, Samsara Therapeutics, Sanofi, Sutro, Tollys, and Vascage. GK is on the Board of Directors of the Bristol Myers Squibb Foundation France. GK is a scientific co-founder of everImmune, Osasuna Therapeu-tics, Samsara Therapeutics and Therafast Bio. GK is in the scien-tific advisory boards of Hevolution, Institut Servier, Longevity Vi-sion Funds and Rejuveron Life Sciences. GK is the inventor of patents covering therapeutic targeting of aging, cancer, cystic fibrosis and metabolic disorders. GK’s brother, Romano Kroemer, was an employee of Sanofi and now consults for Boehringer-Ingelheim. GK's wife, Laurence Zitvogel, has held research con-tracts with Glaxo Smyth Kline, Incyte, Lytix, Kaleido, Innovate Pharma, Daiichi Sankyo, Pilege, Mer-us, Transgene, 9 m, Tusk and Roche, was on the on the Board of Directors of Transgene, is a cofounder of everImmune, and holds patents covering the treat-ment of cancer and the therapeutic manipulation of the microbio-ta.

## AUTHOR CONTRIBUTION

S.F. performed investigations. M.L. and A.S. curated the data and performed formal analyses. O.K visualized results and wrote the first draft of the manuscript. G.K. and O.K. conceptualized the study, supervised, reviewed and edited the manuscript. All au-thors have read and approve the final version of the manuscript.
